# Antithrombotic treatments in patients with acute ischemic stroke and non-valvular atrial fibrillation before introduction of non-vitamin K antagonist oral anticoagulants into practice in Korea

**DOI:** 10.1371/journal.pone.0202803

**Published:** 2018-11-02

**Authors:** Hee-Joon Bae, Ji Hoe Heo, Keun-Hwa Jung, Yong-Seok Lee, Keun-Sik Hong, Woo-Keun Seo, Jaseong Koo, Jae-Kwan Cha, Mi Ji Lee, Bo-Jeong Seo, Young-Joo Kim, Seongsik Kang, Jinmi Seok, Juneyoung Lee, Chin-Sang Chung

**Affiliations:** 1 Department of Neurology, Seoul National University Bundang Hospital, Seoul National University College of Medicine, Seongnam, Korea; 2 Department of Neurology, Severance Hospital, Yonsei University Health System, Seoul, Korea; 3 Department of Neurology, Seoul National University Hospital, Seoul, Korea; 4 Department of Neurology, SMG-SNU Boramae Medical Center, Seoul, Korea; 5 Department of Neurology, Inje University Ilsan Paik Hospital, Goyang, Korea; 6 Department of Neurology, Korea University Guro Hospital, Seoul, Korea; 7 Department of Neurology, Seoul St. Mary’s Hospital, The Catholic University of Korea, Seoul, Korea; 8 Department of Neurology, Dong-A University Hospital, Busan, Korea; 9 Department of Neurology, Samsung Medical Center, Seoul, Korea; 10 Outcomes Research/Real World Data, Corporate Affairs & Health and Value, Pfizer Pharmaceuticals Korea Ltd., Seoul, Korea; 11 Internal Medicine-Medical, Pfizer Pharmaceuticals Korea Ltd., Seoul, Korea; 12 Department of Biostatistics, Korea University College of Medicine, Seoul, Korea; National Cerebral And Cardiovascular Center, JAPAN

## Abstract

**Background:**

This study aimed to describe patterns of long-term antithrombotic use in acute ischemic stroke (AIS) patients with non-valvular atrial fibrillation (NVAF) in Korea and their impacts on clinical events before introduction of non-vitamin K antagonist oral anticoagulants (NOAC) into practice in 2015.

**Methods:**

Patients with NVAF who were admitted due to the AIS and discharged no later than 2008 were enrolled retrospectively. Data were collected at 11 time points during the first 3 years of follow-up. The primary outcome event was a composite of stroke recurrence, major bleeding, and death. Vitamin K antagonist (VKA) users were categorized into a well-controlled INR group and a poorly-controlled INR group (modified TTR ≥47.0% vs <47.0%).

**Results:**

Of 1,350 patients enrolled in this study, 95% were on antithrombotic medications at discharge. The rate of VKA usage decreased over time (77% and 40% at discharge and 3 years, respectively). The cumulative event rates of the primary outcome differed by treatment patterns. Among the 10 most frequent treatment types, the highest outcome rate was observed in patients who started with VKA-only therapy but discontinued VKAs during follow-up without restarting (70.2%); this was followed by those starting with antiplatelet-only therapy and stopping it without restart (66.7%). Among VKA users, the 3-year cumulative primary outcome rates were higher in the poorly-controlled INR group than the well-controlled INR group (24.5% vs 15.7%; *p* = 0.015).

**Conclusion:**

Our study revealed that, in pre-NOAC era, there was a wide spectrum of long-term antithrombotic use. The incidence of the composite outcome also varied by patterns of antithrombotic use.

## Introduction

Atrial fibrillation (AF) confers a 5-fold increase in stroke risk and a 2-fold increase in mortality.[1] Recent studies have reported AF in 20–30% of incident ischemic stroke cases.[2–4] Stroke with AF is more severe and associated with a more substantial societal burden than stroke without AF.[5–8] In a population-based stroke registry study, the case-fatality in patients with AF was shown to reach 50% at one-year after the index stroke.[6]

Current guidelines strongly recommend oral anticoagulant (OAC) therapy for the prevention of recurrent stroke in patients with stroke and AF.[9, 10] Before non-vitamin K antagonist oral anticoagulants (NOAC) were introduced and started to be reimbursed by the Korean government in mid-2015, standard OAC was vitamin K antagonist (VKA). Unpredictable and variable metabolism due to genetic variations and food and drug interactions necessitate close monitoring of the coagulation status of patients using VKA. This has led to the underuse of VKA in practice. According to a recent study of the Registry of the Canadian Stroke Network, one third of patients are not managed according to the recommendations (i.e., OAC alone), although the use of VKA is associated with improved stroke outcomes, even in patients with severe stroke.[11] A study by Riks-Stroke, the Swedish Stroke Register, showed that less than half of ischemic stroke patients with AF maintained VKA treatments during the first 2 years after discharge.[12] However, there has been no report on the long-term adherence to antithrombotic therapy in Korean stroke patients with AF.

The efficacy and safety of VKA are critically dependent on the percentage of the time when International Normalized Ratio (INR) is maintained in time in therapeutic range (TTR).[13–15] However, the TTR might be less than expected in Korean population,[16, 17] possibly due to a high intake of vitamin K-rich foods and high frequency of genetic polymorphisms related to unfavorable VKA metabolism.[18, 19]

This multicenter observational study aimed to describe the 3-year patterns of antithrombotic use before the introduction of NOAC into practice in Korean non-valvular AF (NVAF) patients who were hospitalized due to acute ischemic stroke (AIS). We also investigated the incidence of clinical events, including major bleeding, stroke recurrence, in-hospital mortality, readmission, and emergency room (ER) visits during the 3-year follow-up.

## Methods

### Study design and study population

We designed this retrospective multicenter observational study that was sponsored by BMS/Pfizer Pharmaceuticals Korea Ltd. to explore the real-world practice of antithrombotic use in NVAF patients who were hospitalized due to AIS in Korea. This study conducted in nine university hospitals, scattered nationwide, from July 2014 to March 2016. Through review of the existing stroke registry databases of each center, patients who met the following criteria were identified and enrolled: 1) Patients who were hospitalized due to AIS and discharged on or before December 31, 2008; 2) who had alleged diagnosis or electrocardiographic evidence of AF; 3) who had evidence of relevant ischemic stroke on brain imaging; and 4) who were not enrolled in other interventional clinical studies during the study period (the first three years after discharge). Patients who had prosthetic heart valve or were diagnosed as valvular AF were excluded.

Based on the maximally available resources to conduct this study and the expected precision of ± 2.1% as 95% confidence intervals (CI) with a presumed prevalence of AF in Korean patients with AIS (18.6%) [20], a sample size of 1,350 (150 patients at each participating center) was calculated. Enrollment of study subjects was started from the patient who was hospitalized for AIS in December, 2008 and proceeded retrogradely to reach 150 patients at each center.

### Data collection, follow-up and outcome measures

Clinical data were collected through review of medical records by investigators or trained site staffs at each center. To avoid information bias due to differences in data quality and quality of the existing registry databases among centers, the registry data were used only for screening of the study eligibility.

Time points for data collection were: at discharge, and 1 month (± 2 weeks), 2 months (± 2 weeks), 3 months (± 1 month), 6 months (± 1 month), 9 months (± 1 month), 12 months (± 1 month), 18 months (± 3 months), 24 months (± 3 months), 30 months (± 3 months), and 36 months (± 3 months) after discharge unless patients were lost to follow-up.

Information on the patients’ demographics features (age, sex, body mass index, and date of admission) and clinical characteristics (stroke risk factors, CHADS_2_ scores, CHA_2_DS_2_–VASc scores, and National Institute of Health Stroke Scale [NIHSS] at admission) at the time point of discharge was collected. Information on antithrombotic use (VKA and antiplatelet agents) and clinical events (major bleeding, stroke recurrence, death, readmission, and ER visits) was captured repeatedly at the time points of 1, 2, 3, 6, 9, 12, 18, 24, 30, and 36 months after discharge. The primary outcome measure was defined as a composite of stroke recurrence, major bleeding, and death.

### Modified individual time in therapeutic range

For each patient who received VKA only or VKA plus antiplatelet, INR values were collected at every time point by chart review to assess the quality of anticoagulation. We estimated a point prevalence of optimal INR level control (INR 2.0–3.0) at each time point. A modified iTTR (individual time in therapeutic range)[21,22] was calculated as follows: First, after excluding patients who had less than three times of INR measurements, those who had at least 90 evaluable days within the study period were included for iTTR calculation. The calculation was made by, first, applying the Rosendaal’s linear interpolation method between two consecutive INR measurements;^15^ Second, estimating a patient-specific proportion of INR measurements fall within the value of 2.0 to 3.0 of INR among all of daily-based projected INR measurements between two consecutive measurements dates (including the dates themselves) over the study period; and the lastly, averaging these proportions over study subjects.

### Statistical analysis

For summary statistics, mean (standard deviations, SD) and median (interquartile range IQR) or number of subjects (percentage) were provided for numerical and categorical variables, respectively. For clinical events, the number of events and cumulative 3-month, 6-month, 1-year, 2-year, and 3-year event rates were calculated using the Kaplan–Meier product-limit method.

We assessed the patterns of antithrombotic treatments within three years after discharge (including at the time of discharge). After collecting information on the use of antithrombotic medications, the point prevalence of medication was calculated at each time point. Treatment pattern changes were then categorized as follows: First, the details of antithrombotic medication use in each patient at each time point were examined. Second, we assumed that a patient took the same medication as he/she had taken on his/her last previous visit if there was no record about the medication between recorded visits. If there was no information until the end of follow-up after the last recorded visit, he or she was censored at that visit. Third, the patients’ treatment profiles were categorized based on patterns of medication changes with neglecting distinctions of time point (i.e., visit) and medication-discontinuation gaps between visits. as a result, a total of 26 categories of medication patterns have been identified.

To investigate the effect of INR control on outcomes, VKA users were dichotomized by the median of their modified iTTR’s, and their median survival time was compared using the log-rank test.

All statistical analyses were performed with the SAS software version 9.4 (SAS Institute, Gary, NC USA), and statistical significance was declared when a two-tailed p-value was less than 0.05.

### Ethical approval and patient consent

This study was approved by the institutional review boards of all the participating centers (Seoul National University Bundang Hospital, Severance Hospital, Seoul National University Hospital, SMG-SNU Boramae Medical Center, Inje University Ilsan Paik Hospital, Korea University Guro Hospital, Seoul St. Mary’s Hospital, Dong-A University Hospital and Samsung Medical Center). All procedures have therefore been performed in accordance with the ethics standards of the participating centers and with the 1964 Declaration of Helsinki and its later amendments or comparable ethics standards. Informed consent was waived because of the retrospective nature of the study.

## Results

### Patient characteristics and antithrombotic use

Of the 1,350 patients analyzed in this study, 54% were men and a mean age was 71 years (Table 1). A median NIHSS score at admission was 7, a median CHADS_2_ score was 3.0, and a median CHA_2_DS_2_-VASc score was 5.0. Median follow-up after discharge was 2.33 years (IQR, 0.18–2.97 years) and total follow-up duration is 2,214 person-years. Two-hundred forty-six patients (18.2%) were lost to follow-up after discharge. Comparisons between patients with and without VKA showed that those without VKA were more likely to be old, female, have higher CHADS_2_ and CHA_2_DS_2_-VASc Scores.

**Table 1:** Baseline characteristics of the study population (n = 1,350).

Variables	Total (n = 1,350)	VKA* (n = 1,126)	Non-VKA* (n = 224)	P-value
Male†	729 (54.0)	633 (56.2)	96 (42.9)	0.0002
Age (years)‡	70.8 (64.0–78.0)	70 (64.0–77.0)	77 (70.0–83.5)	< .0001
BMI (kg/m^2^)‡	23.3 (21.0–25.4)	23.5 (21.1–25.5)	22.6 (20.1–24.5)	0.0018
NIHSS at Admission‡	7.0 (2.0–15.0)	6 (2.0–14.0)	12 (4–17)	< .0001
Congestive heart failure†	148 (11.0)	122 (10.8)	26 (11.6)	0.7354
Hypertension†	923 (68.4)	770 (68.4)	153 (68.3)	0.9812
Diabetes mellitus†	345 (25.6)	287 (25.5)	58 (25.9)	0.8992
CHADS_2_ Score†				0.0031
2	223 (16.5)	203 (18.0)	20 (8.9)	
3	489 (36.2)	412 (36.6)	77 (34.4)	
4	475 (35.2)	385 (34.2)	90 (40.2)	
5	150 (11.1)	116 (10.3)	34 (15.2)	
6	13 (1.0)	10 (0.9)	3 (1.3)	
CHA_2_DS_2_-VASc Score†				< .0001
2	63 (4.7)	56 (5.0)	7 (3.1)	
3	201 (14.9)	181 (16.1)	20 (8.9)	
4	310 (23.0)	280 (24.9)	30 (13.4)	
5	363 (26.9)	297 (26.4)	66 (29.5)	
6	293 (21.7)	222 (19.7)	71 (31.7)	
7	106 (7.9)	78 (6.9)	28 (12.5)	
8	12 (0.9)	10 (0.9)	2 (0.9)	
9	2 (0.1)	2 (0.2)		

BMI, body mass index; NIHSS, national institutes of health stroke scale; IQR, interquartile range

P-value for comparing VKA with non-VKA subjects by Student’s t-test or chi-square test, as appropriate

*VKA is patients who have used VKA at least once. Non-VKA is patients who have never used VKA.

† Values are number of subjects (percentage)

‡ Values are median (IQR)

**Fig 1:**
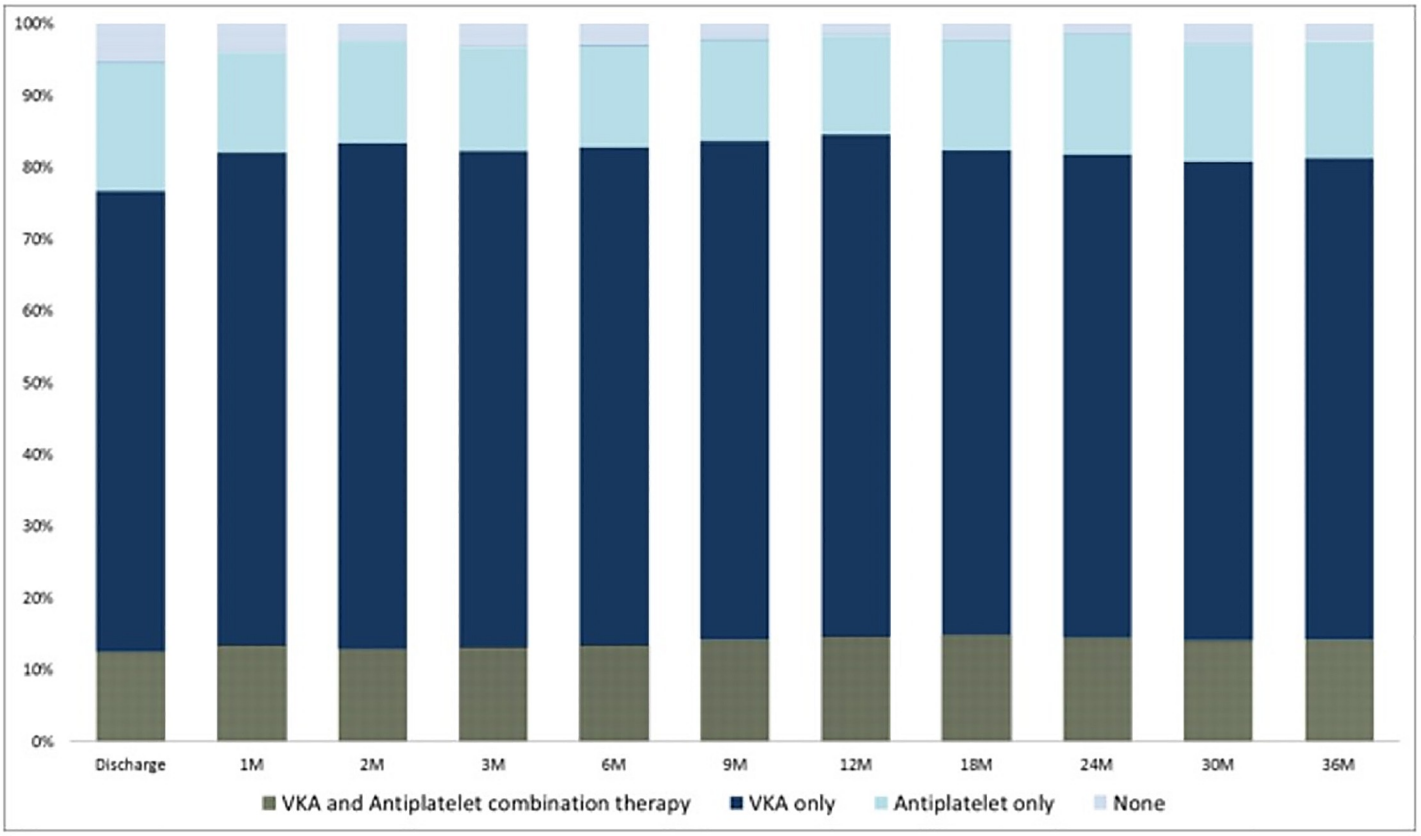
Antithrombotic use at each time point during the 3-year follow-up (Patients with no information were excluded from the denominators.). M indicate month(s); VKA vitamin K antagonists.

At discharge, 95% of patients were on antithrombotic medications; 64% were on VKA only, 18% on antiplatelet drug only, and 12% on both (Table 2). Over time, the proportions of patients being prescribed VKA alone or in combination with antiplatelet agents decreased (77%, 55%, 50%, 45%, and 40% at discharge, 3 months, 1 year, 2 years, and 3 years, respectively). However, when patients with missing information on antithrombotic use were excluded from the denominators, the proportions remained fairly constant (77%, 82%, 85%, 82%, and 81% at discharge, 3 months, 1 year, 2 years, and 3 years, respectively; Fig 1). The proportions of patients being prescribed antiplatelet drugs alone or in combination with VKA were also unchanged (30%, 28%, 28%, 31%, and 30% at discharge, 3 months, 1 year, 2 years, and 3 years respectively).

**Table 2:** Proportion of antithrombotic use at each time point during the 3-year follow-up (before/after excluding missing data from the denominator).

	Discharge	1M	2M	3M	6M	9M	12M	18M	24M	30M	36M
VKA & antiplatelet (%)	12.4 / 12.4	10.8 / 13.5	9.2 / 12.9	8.7 / 13.2	8.3 / 13.4	8.2 / 14.2	8.6 / 14.6	8.8 / 14.8	8.0 / 14.5	7.4 / 14.0	7.0 / 14.1
VKA only (%)	64.2 / 64.2	55.0 / 68.6	50.0 / 70.4	45.9 / 69.1	42.7 / 69.4	40.2 / 69.5	40.9 / 69.9	40.1 / 67.5	37.3 / 67.3	35.4 / 66.8	33.1 / 67.1
Antiplatelet only (%)	18.0 / 18.0	11.2 / 14.0	10.2 / 14.3	9.7 / 14.5	8.7 / 14.2	8.2 / 14.2	8.1 / 13.8	9.1 / 15.4	8.6 / 16.8	8.7 / 16.5	8.0 / 16.3
None (%)	5.3 / 5.3	3.2 / 4.0	1.7 / 2.4	2.5 / 3.2	1.9 / 3.0	1.3 / 2.2	0.9 / 1.6	1.4 / 2.3	0.7 / 1.4	1.5 / 2.7	1.2 / 2.5
Missing* (%)	0	19.9	29.0	33.6	38.4	42.2	41.5	40.7	44.5	47.1	50.6
CHADS_2_≥3/Missing (%)‡		87.7	86.8	85.4	85.3	84.3	83.6	84.9	84.7	84.7	85.1
Death (N)		41	4	12	11	8	23	7	5	7	6
Observed patients at each time point^†^ (N)	1,350	1,350	1,309	1,305	1,293	1,282	1,274	1,251	1,244	1,239	1,232

Values are percentages if not indicated.

M indicate month(s).; VKA, vitamin K antagonists.

* Missing was defined as no information during ± 1 month at 1M, 2M, 3M, 6M, 9M and 12M and during ± 3 month at 18M, 24M, 30M and 36M.

† Number of observed patients at each time point (Calculated by number of total patients minus cumulated number of death at each time point)

‡ Proportions of patients whose CHADS2 scores were 3 or more at discharge among those whose medical records were missing at individual time points

Among antiplatelet drugs, aspirin was most common; about half of patients had aspirin alone or with various combinations (Table 3). Among aspirin users, aspirin monotherapy was a most common type whether they took VKA or not. As expected, antiplatelet polytherapy was more common in the antiplatelet-only group than the VKA plus antiplatelet group. It should be noted that the proportions of patients on antiplatelet monotherapy other than aspirin or clopidogrel was not negligible in both of VKA plus antiplatelet and antiplatelet only groups.

**Table 3:** Types of antiplatelet agents prescribed to patients who were treated with antiplatelet only or VKA plus antiplatelet.

	VKA + antiplatelet				Antiplatelet only			
	Discharge	3M	12M	36M	Discharge	3M	12M	36M
Aspirin + Clopidogrel + Other (%)	-	-	-	1.2	0.4	-	-	1.0
Aspirin + Clopidogrel (%)	8.3	7.0	5.5	3.5	29.2	28.6	25.2	19.1
Aspirin + Other (%)	2.4	0.9	0.9	-	2.1	1.6	1.0	6.1
Clopidogrel + Other (%)	0.6	1.6	1.8	-	1.7	3.2	2.9	2.0
Aspirin (%)	41.7	40.4	37.6	43.0	40.7	31.0	35.0	35.4
Clopidogrel (%)	9.5	8.8	13.8	14.0	12.8	19.8	14.3	29.3
Other antiplatelet monotherapy (%)	37.5	41.2	40.4	38.4	13.2	15.9	11.7	7.1
Observed patients at each time point* (N)	168	114	109	86	243	126	103	99

M indicate month(s); VKA vitamin K antagonists.

* Number of observed patients at each time point (Calculated by number of total patients minus cumulated number of death at each time point)

### Changes in antithrombotic medications and treatment patterns

**Fig 2:**
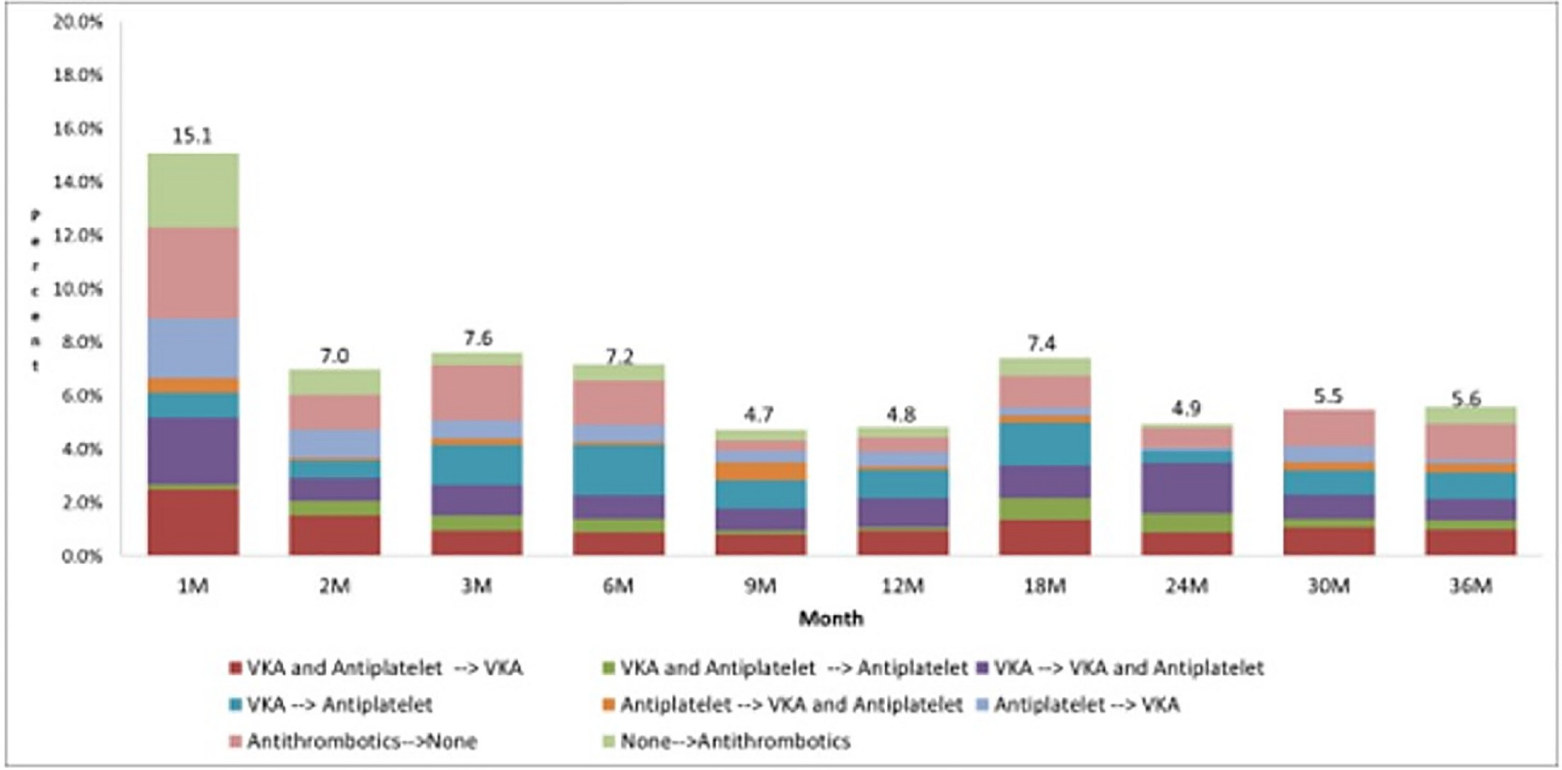
Change of antithrombotic medication at each time point. M indicate month(s); VKA vitamin K antagonists.

We observed the highest rate of change in antithrombotic medications (15%) at 1 month after discharge (Fig 2). Thereafter, change rates were stabilized (5–8% of patients). During the first 3 and last 6 months of follow-up, the most common change was a discontinuation of antithrombotic medications (1.3–3.4% of patients). In the intermediate period, the most common change was from VKA to antiplatelet drugs (1.1–1.9% of patients).

**Fig 3:**
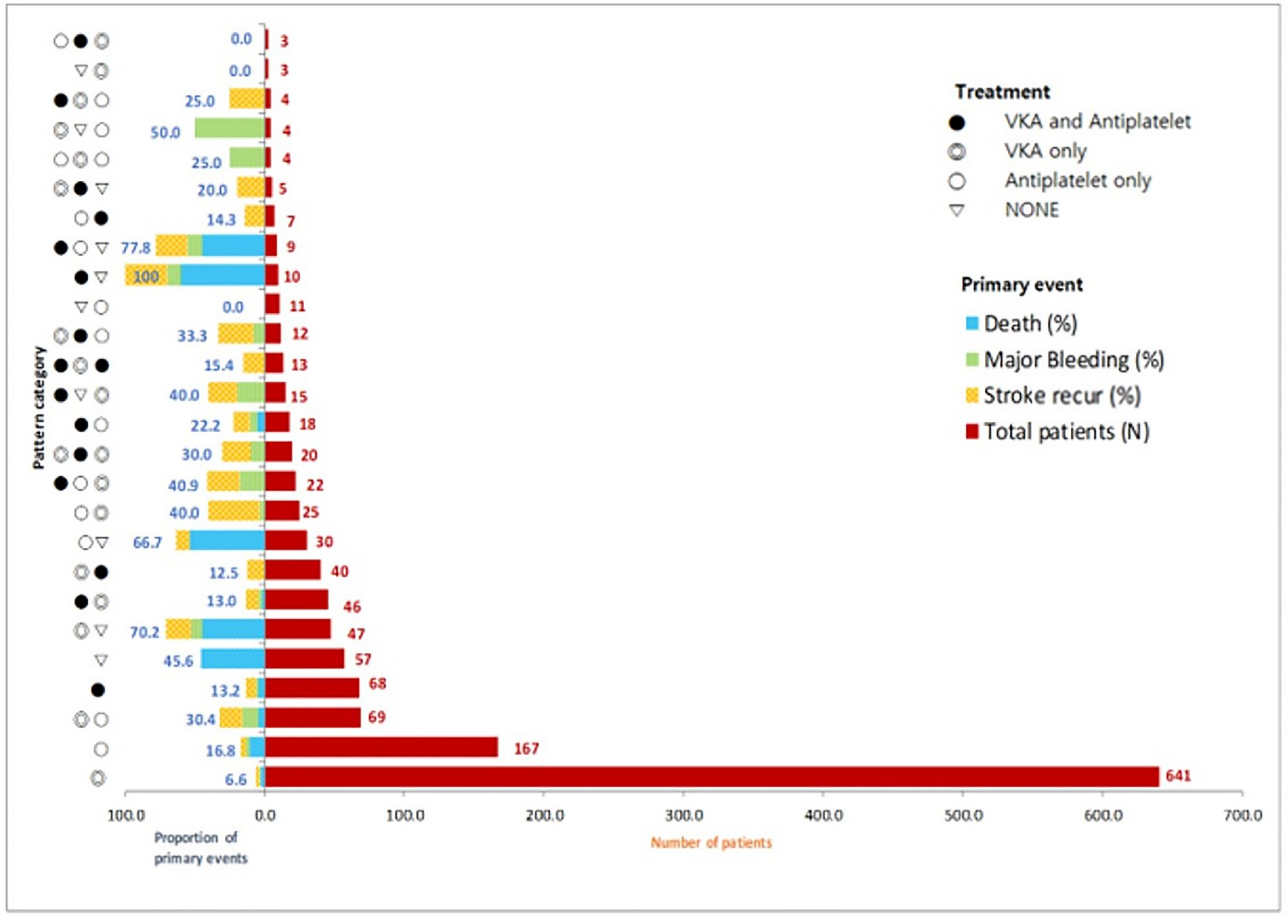
Number of patients according to treatment patterns and proportions who experienced primary outcome events according to the types (The definitions of treatment patterns are provided in Table 4.). M indicate month(s); VKA vitamin K antagonists. *Note. If there is one symbol in the pattern category, this means that only one treatment is maintained. If there are two or more symbols in the pattern category, this category indicates that the treatment was changed.

We then analyzed treatment patterns within the 3-year follow-up period. The 531 patterns that were initially identified were reduced to 387 after imputing no record of medication prior to the patient’s last visit of 36 months with his/her last observed medication information. Further disregard of time point distinction and medication-discontinuation gap between visits resulted in a total of 26 treatment pattern categories (Fig 3). The rules defining these 26 categories are provided in Table 4. Maintaining VKA-only therapy during follow-up was the most common treatment pattern (47.5%), followed by maintaining antiplatelet-only (12.4%), VKA only (5.1%), and maintaining VKA therapy in combination with antiplatelet agents (5.0%).

**Table 4:** Rules for defining treatment patterns.

**1. Rules for dealing with missing data on the pharmaceutical prescriptions (531 patterns before rule application/ 387 patterns after rule application)**													
1)	Applicable to all prescriptions 1-1) In a case where a medication prescription record was unavailable for a specific follow-up time, and the prescription records for the preceding and following follow-up time were available, the prescription at the specific follow-up time was presumed as the same as that received at the preceding follow-up time. 1-2) If there were no the prescription records after the last prescription, the missing data was not redefined. (Ex.) In a case where at 9 and 18 months after discharge, prescriptions for medication were available, and at 12 months after discharge, medication prescription was unavailable, the prescribed medication at 12 months after discharge was defined as the same as that prescribed at 9 months after discharge.												
	(Ex 1) Pattern ID 87												
	① Before application												
	Pattern ID	No. of patients	Discharge	1M	2M	3M	6M	9M	12M	18M	24M	30M	36M
	87	2	◎	◎		◎	◎	◎	◎				
	② After application												
	Pattern ID	No. of patients	Discharge	1M	2M	3M	6M	9M	12M	18M	24M	30M	36M
	87	2	◎	◎	◎	◎	◎	◎	◎				
	*M indicates month(s)*.												
2)	Applicable to 'VKA & Antiplatelet' or 'VKA only' groups 2–1) If a prescription at discharge was 'VKA & Antiplatelet' or 'VKA only' and there were no prescription records for the next two follow-up times with a prescription available at 3 months after discharge, the missed records at 1 month and 2 months, which were within 3 months after discharge, were regarded as the same as the prescription record at discharge.												
	(Ex 2) Pattern ID 29												
	① Before application												
	Pattern ID	No. of patients	Discharge	1M	2M	3M	6M	9M	12M	18M	24M	30M	36M
	29	5	◎			◎	◎	◎	◎	◎	◎	◎	◎
	② After application												
	Pattern ID	No. of patients	Discharge	1M	2M	3M	6M	9M	12M	18M	24M	30M	36M
	29	5	◎	◎	◎	◎	◎	◎	◎	◎	◎	◎	◎
	*M indicates month(s)*.												
3)	Applicable to 'Antiplatelet only' groups In a case where a prescription at 2 months after discharge was ‘antiplatelet only’ and there were no prescription records at 3 months and 6 months after discharge, and the prescription record at 9 months after discharge was available, the missed data at 3 and 6 months were defined as ‘antiplatelet only’.												
	(Ex 3) Pattern ID 29												
	① Before application												
	Pattern ID	No. of patients	Discharge	1M	2M	3M	6M	9M	12M	18M	24M	30M	36M
	29	5	◎		○			●		○			
	② After application												
	Pattern ID	No. of patients	Discharge	1M	2M	3M	6M	9M	12M	18M	24M	30M	36M
	29	5	◎	◎	○	○	○	●	●	○			
	*M indicates month(s)*.												
**2. Additional rules for treatment pattern simplification (203 patterns after rule application)**													
1)	If no prescription records were at the midpoint of the follow-up, the missed data were completed with prescription records at the preceding follow-up time.												
2)	If there were no prescription records before completion of the follow-up period after the last prescription record, missing data up to 36 months were completed by using the last prescription record.												
	(Ex) Pattern ID 270												
	① Before application												
	Pattern ID	No. of patients	Discharge	1M	2M	3M	6M	9M	12M	18M	24M	30M	36M
	270	1	●	●	●	◎			◎	◎			
	② After applicating the rule of No. 2–1)												
	Pattern ID	No. of patients	Discharge	1M	2M	3M	6M	9M	12M	18M	24M	30M	36M
	270	1	●	●	●	◎	◎	◎	◎	◎			
	② After applicating the rule of No. 2–2)												
	Pattern ID	No. of patients	Discharge	1M	2M	3M	6M	9M	12M	18M	24M	30M	36M
	270	1	●	●	●	◎	◎	◎	◎	◎	◎	◎	◎
	*M indicates month(s)*.												
**Rules 1 and 2 were applied to correct all the prescription patterns without missing data from discharge time to 36 months after discharge. Finally, a total of 26 treatment patterns were generated.**													

### Incidence of the primary outcome

**Fig 4:**
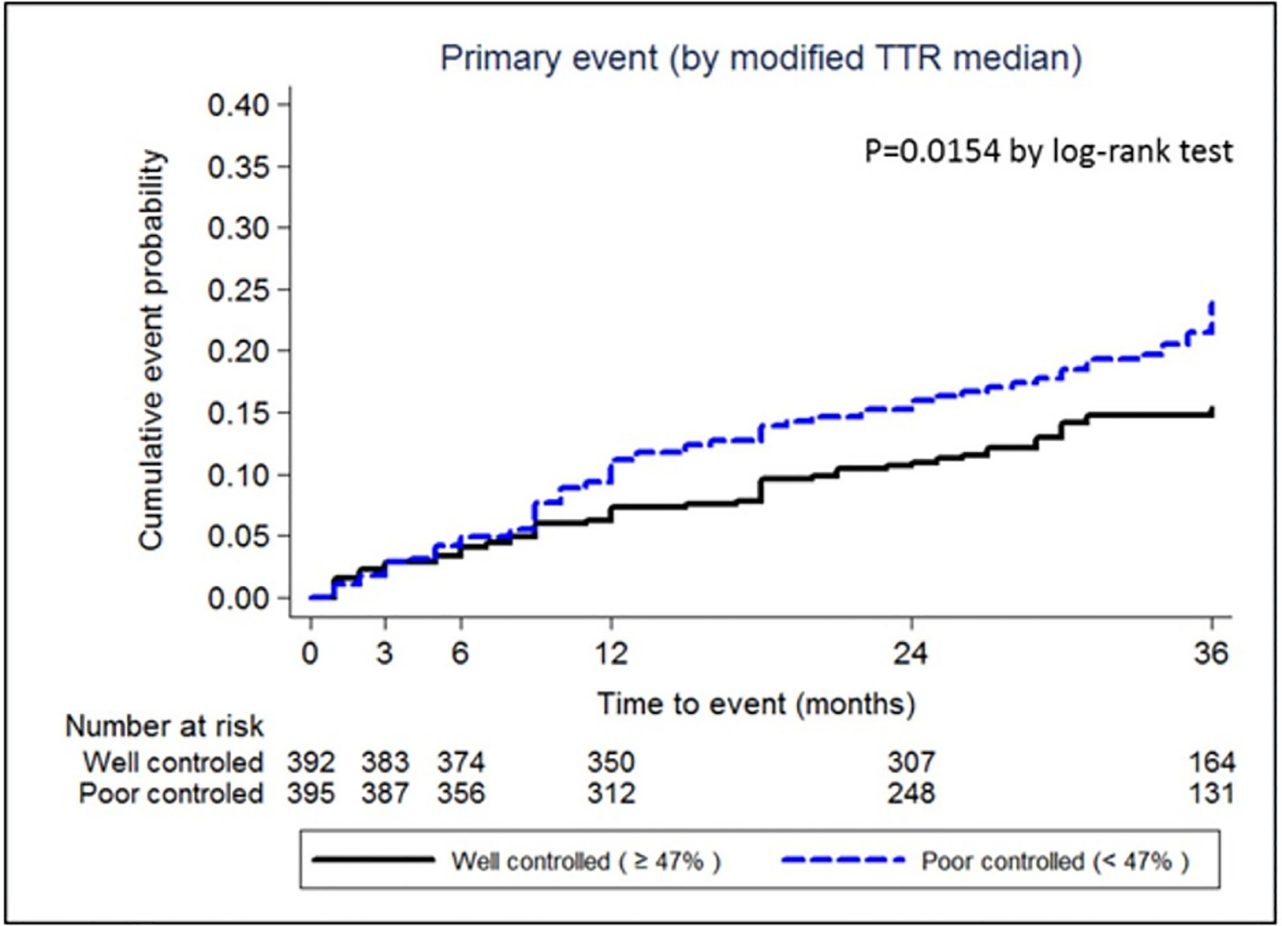
Cumulative incidence curve for clinical events.

The primary outcome event (a composite of stroke recurrence, major bleeding, and death) occurred in 254 of the 1,350 patients during the follow-up period. The cumulative event rate at 3 years was 27.0% (95% CI, 24.0–30.0%; Fig 4). One-hundred six strokes, 48 major bleedings, and 124 deaths occurred. Among 106 strokes, 67 were ischemic, 25 hemorrhagic and 18 undetermined. The 3-year cumulative event rates of stroke recurrence, major bleeding, and death were 13.1% (95% CI, 10.7–15.6%), 5.5% (95% CI, 4.0–7.1%), and 13.7% (95% CI, 11.3–16.0%), respectively. Readmission occurred in 120 patients with a 3-year cumulative event rate of 13.6% (95% CI, 11.3–16.0%). A total of 80 patients visited the ER within the follow-up period, with a 3-year cumulative rate of 9.6% (95% CI, 7.6–11.7%). Annualized incidence rates of primary outcome events, each component of primary outcome measure, re-admission, and ER visit are presented in Table 5.

**Table 5:** Incidence rates of outcome events.

	Number of events	Incidence rate per 100 person-years	95% CI
Primary events	254	11.47%	10.06–13.96%
Major bleeding	48	2.17%	1.55–5.08%
Stroke recur	106	4.79%	3.88–7.62%
Death	124	5.60%	4.61–8.40%
Re-admission	120	5.42%	4.45–8.22%
ER visit	80	3.61%	4.45–8.22%

The 3-year cumulative event rates of the primary outcome differed according to the treatment patterns. It was highest in patients who started with VKA therapy in combination with antiplatelet drugs but who discontinued therapy during follow-up and did not take any antithrombotic medications until the end of follow-up (100%; Fig 3). This was followed by those who started with VKA therapy in combination with antiplatelet drugs, changed to antiplatelet-only, and discontinued it after then (78%). Among the 10 most frequent types, the highest rate was observed in patients who starting with VKA-only therapy and discontinued it without restart mid-follow-up (70.2%). The second highest rate was seen in patients who started with antiplatelet-only therapy and discontinued it without restart (66.7%). Among treatment types with no change in antithrombotic medication, the 3-year cumulative event rates of the primary outcome ranged between 6.6% and 16.8%; these rates were lower than the rates observed for treatment types with changes in antithrombotic medications.

### International normalized ratios and primary outcomes by modified iTTR

**Fig 5:**
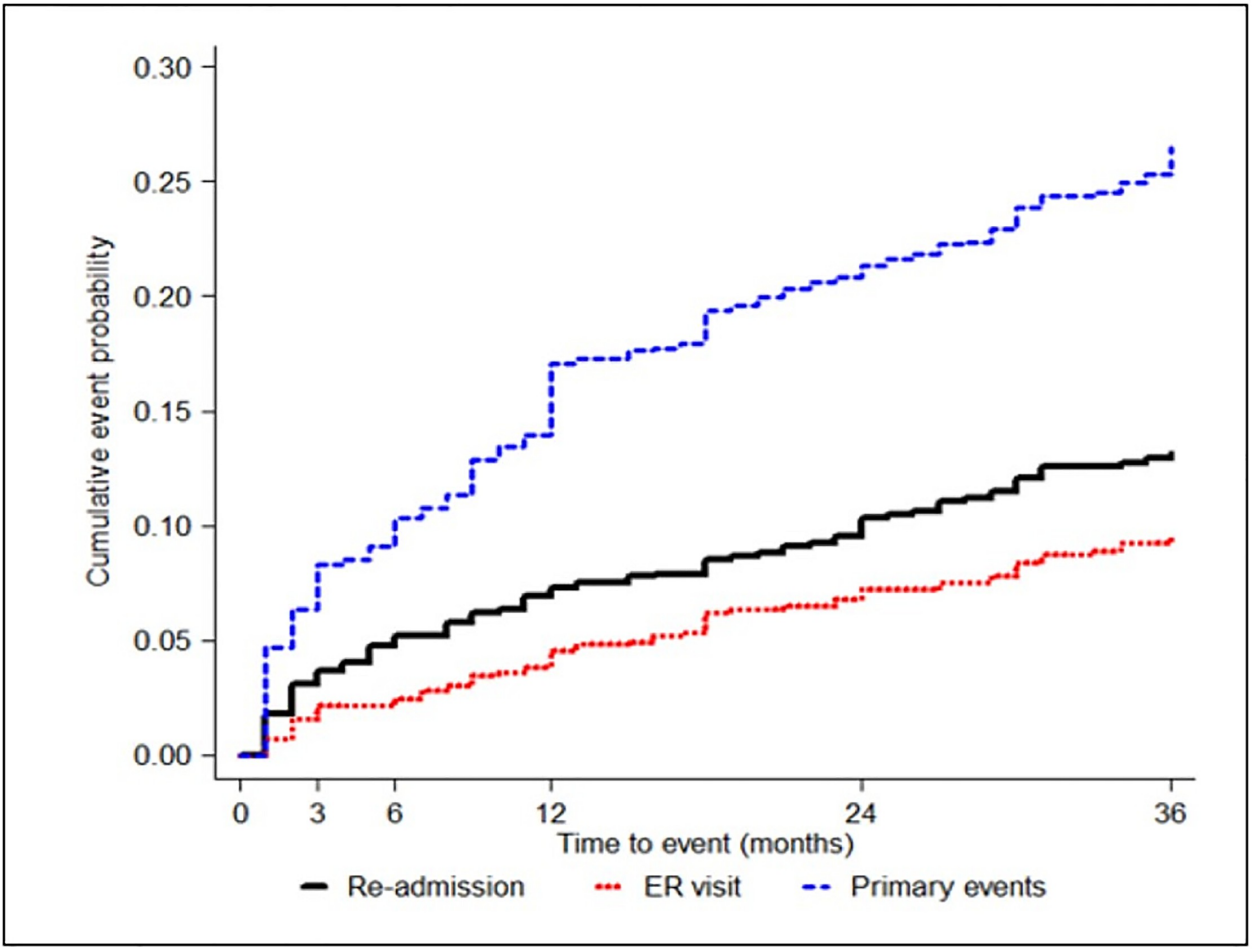
Point prevalence of optimal INR levels in the VKA only and the VKA plus antiplatelet groups at each time point. M indicate month(s).

A total of 1,253 patients had one or more INR measurement(s) during follow-up (mean number of INR measurements: 5.61). The point prevalence of optimal INR (2–3) in patients taking VKA alone or in combination with antiplatelet drugs (n = 1,028) was highest at discharge (586 of 1028, 57.0%), lowest at 2 months after discharge (209 of 564, 37.1%) and then increased slowly until the end of follow-up (Fig 5). A median modified iTTR was 46.9% (IQR, 27.3–64.3%) and a mean modified iTTR was 45.6% (SD, 25.7%). The proportion of patients who achieved modified iTTR >60% was 31.4%.

**Fig 6:**
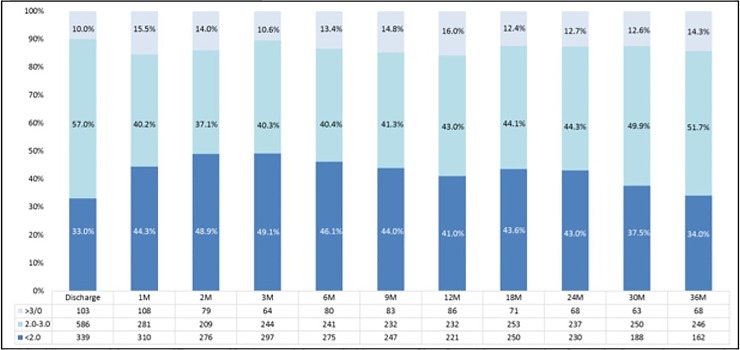
Primary outcome events according to modified iTTR. iTTR, individual time in therapeutic range.

VKA users were then categorized into a well-controlled INR group and a poorly-controlled INR group (modified iTTR ≥47.0% *vs*. <47.0%). The 3-year cumulative primary outcome rates were 15.7% and 24.5% in the well- and poorly-controlled groups, respectively; the difference was statistically significant (*p* = 0.015; Fig 6).

## Discussion

Of the 1,350 patients who were hospitalized due to AIS and had AF in our study, 77% were discharged with VKA and this proportion decreased over time (40% at 3 years). We could find diverse patterns of antithrombotic use during 3 years after stroke. Primary outcome rates differed by these patterns, and discontinuation of antithrombotic agents, especially VKA, seemed to be associated with primary outcome. Among the VKA users, the poorly-controlled INR was at higher risk for primary outcome than the well-controlled INR group.

A multicenter registry-based study that examined the patterns of antithrombotic use in AIS patients with AF in the United States showed that 87% were on OAC therapy at discharge.(8) Another multicenter registry-based study in Canada reported an OAC prescription rate of 70% at discharge.[11] It should be noted that the enrollment periods of the study subjects in these three studies (including ours) were 2008 or earlier. A recent multicenter-registry study in Japan, which enrolled study subjects between 2011 and 2014, reported a much higher OAC prescription rate at discharge (96%); VKA was prescribed in 56% and NOACs in 40%.[23] The use of NOACs in the treatment of AF was officially allowed between 2011 and 2013 in Korea (dabigatran in March 2011, rivaroxaban in April 2012, and apixaban in February 2013), but it was limitedly allowed to patients who experienced bleedings or ischemic events during taking VKA or had contra-indications for VKA. In 2015, indications for NOACs were expanded like other developed countries and an increase in the use of NOAC is expected.

The OAC prescription rate decreased over time in our study (77% at discharge, 50% at 1 year, 45% at 2 years, and 40% at 3 years). Data from the Adherence eValuation After Ischemic Stroke Longitudinal (AVAIL) registry in the United States showed an OAC prescription rate of 87% at discharge and 78% at 1 year after discharge.[8] In the Riks-Stroke registry of Sweden, rates were 89%, 65%, and 45% at 4 months, 1 year, and 2 years, respectively.[12] The proportions of patients who were excluded from analysis due to lack of follow-up data at the last follow-up were 6.0% in the AVAIL registry and about 1% in the Riks-Stroke Registry; in contrast, it was 50% in our study. This higher proportion of missing information in our study may be attributed to collection of data by retrospective chart review and years of time gaps between patient visits at out-patient clinic and data collection. When we excluded patients with missing follow-up data from the denominators, OAC prescription rates were relatively constant during the 3-year follow-up period, ranging between 81% and 83%. True OAC prescription rates seem to exist between them; a study linking data from the registry and national insurance claim databases might provide answers.

Antiplatelet drugs alone or in combination with VKAs were given to 30% of patients at discharge. This rate was relatively constant during the 3-year follow-up period; it is significantly lower than the rates reported in Western countries: 47% and 46% at discharge and at 1 year, respectively, for the AVAIL Registry[8] and 53% at discharge for the Ontario Stroke Registry of Canada.[11] This difference could be explained by differences in the proportions of patients who were being prescribed VKAs in combination with antiplatelet agents. The rate ranged between 12% and 14% in our study, whereas it was 31% in the Ontario Stroke Registry and 36% in the AVAIL Registry. The lower rates in our study can be explained, at least partly, by the lower prevalence of coronary heart disease (CHD) in Korean ischemic stroke patients with AF. We could not gather information on CHD in this study; however, according to a recent report from Korea, the CHD prevalence in AIS patients with AF was 16%;[24] in contrast, it was 37% in the AVAIL Registry.

As expected, aspirin was a most commonly prescribed antiplatelet drug in patients taking VKA in combination with antiplatelet agents in our study (52% and 44% at discharge and 1 year, respectively) as well as in those on antiplatelet-only therapy (72% and 61% at discharge and 1 year, respectively). In the AVAIL registry, aspirin was prescribed to 95% and 98% of patients taking VKA in combination with antiplatelet agents at discharge and 1 year, respectively; and to 85% and 89% of patients taking antiplatelet drugs only at discharge and 1 year, respectively.[8] The use of antiplatelet drugs other than aspirin seems to be higher in our study when compared to the AVAIL registry.

To the best of our knowledge, this study might be the first one to report changes in antithrombotic medication patterns during a long-term follow-up period in AIS patients with AF. Treatment patterns were more complex than expected (Fig 3). During the first 3 years after stroke, 69% of patients did not change antithrombotic medications significantly. The remaining 31%, however, changed treatment heavily; most common patterns were stopping OAC without restart (17%). Fig 3 may suggest an association between discontinuation of antithrombotic medications without restart and occurrence of clinical events, but it should be interpreted cautiously and confirmed by a well-designed prospective study in the future.

We found a mean modified iTTR of 45.6% in this study; the proportion of patients who achieved a modified iTTR >60% was 31.4%. In pivotal NOAC trials, the mean TTR of Korean patients randomized to warfarin was much lower than the average TTR of overall study populations.[16, 17, 25, 26] This study confirms that TTR in practice might be much lower than that in clinical trial settings in Korea. A Korean single center study reported a TTR of 57.5% in AF-related stroke patients whose warfarin therapy was managed by a formal anticoagulation clinic.[27] As expected, patients with higher modified iTTRs had lower rates of primary outcome events than those with lower modified iTTRs.

This study has several limitations. First, most of the participating centers are large tertiary hospitals; therefore, the study subjects might be not representative of the Korean stroke population with AF. Second, underestimations of outcome events and biases due to inadequate information might have been inevitable as most data on antithrombotic medications, INRs, and outcome events were based on retrospective review of medical records. Third, consensus about the classification of long-term antithrombotic treatment patterns was lacking. The arbitrariness of our classification might affect the generalization of our results. Fourth, although lost to follow-up and missing information were unavoidable in this retrospective study, the attrition rate of 50% in our study was higher than expected. Thus, caution should be exerted when trying to generalize our results. Fifth, we could not collect information on reasons why antithrombotic medications were stopped or changed. Therefore, we could not exclude that backgrounds of changing treatment differed even within the same treatment patterns: for example, one stopped VKA due to serious bleeding but another did due to poor compliance.

Despite these limitations, our study, to the best of our knowledge, was the first to explore antithrombotic treatment patterns throughout a long-term follow-up period after AIS in Korean patients with AF. Our study revealed that before the introduction of NOACs in Korea in 2015, <80% of AIS patients with AF were discharged with OAC therapy; thereafter, the OAC prescription rate seemed to gradually decrease. When compared to other Western countries, antiplatelet drugs were used less frequently in our study population; this can be mostly explained by the less frequent use of antiplatelet agents in combination with VKA. The lower prevalence of CHD in the Korean population might be an explanation. Third, aspirin was the most common antiplatelet drug; however, the use of antiplatelet medications other than aspirin was more frequent than expected. Fourth, we observed complex patterns of antithrombotic treatment during the 3-year follow-up period. Antithrombotic medications were often discontinued without being restarted; this might have been associated with clinical events. Fifth, INR control status in VKA users was poorer in practice than expected (achieved modified iTTR >60% of 31.4%). A future prospective study should elucidate the clinical meanings and consequences of these complex treatment patterns.

## Supporting information

S1 Appendix Data of Antithrombotics in AIS with NVAF patients. (XLSX)

S1 Table Supplement patterns. (XLSX)
